# Editor’s Book Table

**Published:** 1870-07

**Authors:** 


### Editor's Book Table.

[Note.—All works reviewed in the columns of the Chicago Medical Journal may be found in the extensive stock of W. B. Keen & Cooke, whose catalogue of Medical Books will be sent to any address upon request.]

*Renal Diseases:* A Clinical Guide to their Diagnosis and Treatment. By W. R. Basham, M.D., Fellow of the Royal College of Physicians; Senior Physician to the Westminster Hospital, and Lecturer on Medicine, etc., etc. With Illustrations. Philadelphia: Henry C. Lea, 1870. Pp. 304.

This is an unpretending volume, but one which we take pleasure in recommending to our readers as a clear and practical essay—one which will materially facilitate the acquisition of practical clinical ability, both with regard to diagnosis and treatment. It is divided into three parts; the first of which is devoted to the various forms of Nephritis, their symptoms, diagnosis and treatment; the second discusses "Chronic Nephritis—Chronic Albuminuvia," or those diseases without primary symptoms of inflammation; the third reviews, with illustrations, the Clinical Significance of the Urine.

The ten pages on Tubercular Nephritis and Pyelitis are, alone, worth more than the price of the entire volume. It is the most satisfactory exposition of the subject in a short space, that we have had the fortune to meet.

*Gray's Anatomy*,—

The new edition of which was announced in the June number, needs no introduction to our readers. It is pre-eminently *the* text book. This edition comprises much valuable new matter upon the topics: general anatomy, the development of the ovum and fœtal structures, and a compendious introduction to the study of microscopic anatomy. We scarcely see how the book can be further improved for the student. All else wanting can be furnished only by the scalpel and cadaver.

*United States Sanitary Commission Memoirs* — Surgical — receipt acknowledged last number, is a volume uniform with the Medical Memoirs previously issued. Octavo, pp. 586, and ten chromo lithograph plates. $6.50.

The first memoir treats: "On the Wounds of Bloodvessels, Traumatic Hemorrhage, Traumatic Aneurism, and Traumatic Gangrene." The second: "On the Secondary Traumatic Lesions of Bone, namely: Osteo-Myelitis, Periostitis, Ostitis, Osteo-Porosis, Caries, and Necrosis." The third: "Pyæmia."

The memoirs are by John A. Lidell, A.M., M.D., etc., etc. Edited by Prof. Frank Hastings Hamilton.

A large amount of valuable material has been utilized to an extent which we had reason to anticipate from the high reputation both of the author and editor.

